# Genomic Fossils Calibrate the Long-Term Evolution of Hepadnaviruses

**DOI:** 10.1371/journal.pbio.1000495

**Published:** 2010-09-28

**Authors:** Clément Gilbert, Cédric Feschotte

**Affiliations:** Department of Biology, University of Texas, Arlington, Texas, United States of America; Fred Hutchinson Cancer Research Center, United States of America

## Abstract

Because most extant viruses mutate rapidly and lack a true fossil record, their deep evolution and long-term substitution rates remain poorly understood. In addition to retroviruses, which rely on chromosomal integration for their replication, many other viruses replicate in the nucleus of their host's cells and are therefore prone to endogenization, a process that involves integration of viral DNA into the host's germline genome followed by long-term vertical inheritance. Such endogenous viruses are highly valuable as they provide a molecular fossil record of past viral invasions, which may be used to decipher the origins and long-term evolutionary characteristics of modern pathogenic viruses. Hepadnaviruses (Hepadnaviridae) are a family of small, partially double-stranded DNA viruses that include hepatitis B viruses. Here we report the discovery of endogenous hepadnaviruses in the genome of the zebra finch. We used a combination of cross-species analysis of orthologous insertions, molecular dating, and phylogenetic analyses to demonstrate that hepadnaviruses infiltrated repeatedly the germline genome of passerine birds. We provide evidence that some of the avian hepadnavirus integration events are at least 19 My old, which reveals a much deeper ancestry of Hepadnaviridae than could be inferred based on the coalescence times of modern hepadnaviruses. Furthermore, the remarkable sequence similarity between endogenous and extant avian hepadnaviruses (up to 75% identity) suggests that long-term substitution rates for these viruses are on the order of 10^−8^ substitutions per site per year, which is a 1,000-fold slower than short-term rates estimated based on the sequences of circulating hepadnaviruses. Together, these results imply a drastic shift in our understanding of the time scale of hepadnavirus evolution, and suggest that the rapid evolutionary dynamics characterizing modern avian hepadnaviruses do not reflect their mode of evolution on a deep time scale.

## Abstract 2

Ancient hepadnavirus sequences found in bird genomes reveal that this family of viruses, which includes the human hepatitis B virus, is much older than previously thought.

## Author Summary

Paleovirology is the study of ancient viruses and the way they have shaped the innate immune system of their hosts over millions of years. One way to reconstruct the deep evolution of viruses is to search for viral sequences “fossilized” at different evolutionary time points in the genome of their hosts. Besides retroviruses, few virus families are known to have deposited molecular relics in their host's genomes. Here we report on the discovery of multiple fragments of viruses belonging to the Hepadnaviridae family (which includes the human hepatitis B viruses) fossilized in the genome of the zebra finch. We show that some of these fragments infiltrated the germline genome of passerine birds more than 19 million years ago, which implies that hepadnaviruses are much older than previously thought. Based on this age, we can infer a long-term avian hepadnavirus substitution rate, which is a 1,000-fold slower than all short-term substitution rates calculated based on extant hepadnavirus sequences. These results call for a reevaluation of the long-term evolution of Hepadnaviridae, and indicate that some exogenous hepadnaviruses may still be circulating today in various passerine birds.

## Introduction

Most viruses are characterized by high substitution rates, which generally prevent reconstruction of their long-term evolutionary history [1]. Consequently, the origins and age of most extant viruses remain elusive [2]. One solution to this conundrum lies in the advent of paleovirology, the study of paleoviruses and the way they have shaped the antiviral genes of their hosts over millions of years [3]. Although viruses lack a true geological fossil record, some have left footprints of their evolution in their hosts' genome. For example, vertebrate retroviruses are RNA viruses that normally integrate into the genome of their host's somatic cells as part of their replication cycle. On occasion, these viruses may integrate into the germline genome of their host, and become inactive and vertically inherited over millions of years. Their molecular relics, called endogenous retroviruses, now make up a substantial fraction of vertebrate genomes (∼8% in human; [4]).

While retroviruses account for the major fraction of known viral genomic fossils, various other viruses that do not normally integrate into the genome but replicate in the nucleus of the host cell are susceptible to fortuitous chromosomal integration. For example, pararetroviruses (double-stranded DNA) have deposited numerous endogenous copies in the genome of several plant species [5], and singular integration events have been reported for gemini-like viruses (single-stranded DNA) in tobacco [6], and for non-retroviral RNA viruses such as totovirus-like and M2-killer-like viruses in fungi (double-stranded RNA; [7],[8]) and flaviviruses in mosquitoes [9],[10].

Genomic fossils closely related to modern viral groups are of particular interest as they have the potential to unveil otherwise inaccessible features of the long-term evolution of viruses. A handful of such precious paleoviruses have recently been unearthed from mammalian genomes. Among these, two ancient lentiviruses (RELIK in rabbit [11] and pSIV in primates [12],[13]) and one foamy virus (SloEFV in xenarthrans [14]) revealed that the history of these two retroviral genera can be rooted on a deep time scale, challenging earlier views on retroviral evolution based on comparisons of extant viral genomes. Likewise, the recent discovery of multiple endogenous bornaviruses and filoviruses in diverse mammals showed that these single-stranded RNA viruses were able to infiltrate repeatedly the germline of distant mammalian species over at least the past 40 My [15]–[17].

Hepadnaviridae (including hepatitis B viruses [HBVs]) are compact (∼3,000 bp), partially double-stranded circular DNA viruses infecting various mammal and bird species and responsible for ∼600,000 human deaths of acute or chronic liver disease per year [18]. While replication of these viruses does not rely on integration into the host genome, a relatively large number of chromosomal integration events have been characterized in mammalian liver cells sampled from chronically infected individuals [19]. In this study, we show that hepadnaviruses have also infiltrated the germline genome of some of their vertebrate hosts in the distant past. The viral sequences fossilized since these endogenization events offer an unprecedented opportunity to reevaluate the mode and tempo of Hepadnaviridae evolution.

## Results

### Endogenous Hepadnaviruses in the Zebra Finch Genome

**Figure 1:**
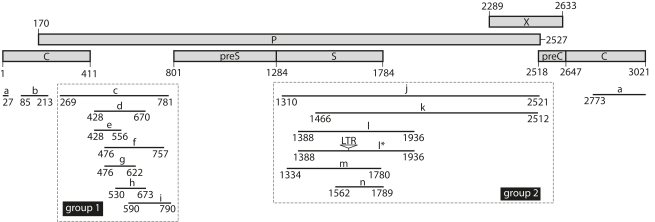
Map showing the position of the eZHBVs in the DHBV genome. Grey rectangles represent the different open reading frames of DHBV (GenBank accession number AY494851, isolated from a puna teal) encoding the precore (preC), core (C), polymerase (P), surface (preS and S), and regulatory HBx-like (X) proteins. eZHBVs (black lines) are labeled with letters as in Table 1. Group 1 and 2 correspond to the two groups of overlapping fragments used for the phylogenetic analyses (Figure 2). Asterisks indicate the occurrence of segmental duplication (see text). The white triangle in eZHBVl* indicates the presence of a 618-bp solo LTR belonging to the TguLTRK1b LTR retrotransposon family [41]. The solo LTR is flanked by a 6-bp target site duplication (GCTCTC).

TBLASTN searches using the duck HBV (DHBV) proteins on all available genomes in GenBank yielded 15 hepadnavirus-like fragments (collectively called endogenous zebra finch HBVs [eZHBVs]). These sequences are interspersed into ten different chromosomes of the zebra finch (*Taeniopygia guttata*, Estrildidae) and show between 55% and 75% nucleotide similarity to the DHBV genome (Figure 1; Table 1; Dataset S1). Most of these fragments contain one or more mutations compromising their coding capacity, which suggests that they have evolved under no functional constraint since integration. Together, the 15 eZHBV segments cover ∼70% of the DHBV genome, which is structurally representative of all hepadnaviruses [20] (Figure 1). eZHBVs tend to map within two loosely defined regions of DHBV, one encompassing the core and polymerase N-terminal domains (eZHBVc–eZHBVi; group 1), and one overlapping with the preS/S and polymerase C-terminal domains (eZHBVj–eZHBVn; group 2). In addition, two eZHBVs (eZHBVa and eZHBVb) map to other regions of the core domain (Figure 1). eZHBVl and eZHBVl* (both located on Chromosome 20) map to the same region of the DHBV genome and are highly similar (97% over 537 bp). Similar levels of identity are observed between their flanking genomic regions: 96.7% identity over 637 bp in the 5′ flanking region and 97% identity over 534 bp in the 3′ flanking region. These observations suggest that one insertion most likely derives from the other through intrachromosomal duplication of a genomic fragment including the initial eZHBV insertion along with its flanking regions.

**Table 1:** Characteristics of the endogenous HBV fragments found in the zebra finch genome (eZHBVs).

eZHBV	Position in DHBV^a^		Position in Finch Genome			Length (bp)	NS Mutations^b^	Similarity to DHBV	5′ Gene in Finch	3′ Gene in Finch
	Start	End	Chromosome	Start	End					
a	2773	27	24	5,223,682	5,223,949	268	0/1	58.9%	Scn3b (690 bp^c^)	Scn3b (50 bp)
b	85	213	1	50,694,672	50,694,800	129	1/0	63.8%	Fry (2.2 kb)	Fry (1.7 kb)
c	269	781	12	3,002,393	3,002,898	507	1/0	61.5%	ATP2B2 (10 kb)	ATP2B2 (42 kb)
d	428	670	26	864,217	864,459	246	1/0	59.3%	Trim33 (1.3 kb)	Trim33 (12.5 kb)
e	428	556	6	2,324,466	2,324,594	129	0/0	62%	cdh23 (5.5 kb)	cdh23 (500 bp)
f	476	757	1A	68,942,470	68,942,804	335	4/1	62.2%	LMO3 (13 kb)	MGST1 (34 kb)
g	476	622	1A	68,944,006	68,944,152	147	1/1	70.5%	LMO3 (15 kb)	MGST1 (32 kb)
h	530	673	8	25,658,020	25,658,163	144	0/0	69.4%	—	—
i	590	790	Z	8,647,882	8,648,070	185	1/0	60.8%	—	—
j	1310	2521	Z	8,648,446	8,649,654	1,209	1/0	65.5%	—	—
k	1466	2512	1	50,694,887	50,695,943	1,057	4/5	54.8%	Fry (2.5 kb)	Fry (500 bp)
l	1388	1936	20	1,111,838	1,112,362	537	2/0	72.3%	—	C20orf4 (14 kb)
l*	1388	1936	20	1,119,136	1,120,278	537	1/0	72.7%	—	C20orf4 (6 kb)
m	1334	1780	6	33,336,955	33,337,390	436	2/3	75%	Dhx32 (1 kb)	Dhx32 (100 bp)
n	1562	1789	19	5,856,561	5,856,833	273	0/0	72.8%	—	—

eZHBVa–eZHBVe, eZHBVk, and eZHBVm are in an intron; a dash indicates that there is no gene within 50 kb 5′ and/or 3′ of the insertion. eZHBVl and eZHBVl* derive from post-insertional duplication (see Results for details). Positions in the zebra finch genome are from the UCSC Genome Bioinformatics browser, based on the assembly WUGSC 3.2.4/taeGut1 (July 2008). See Figure 1 for the mapping of eZHBVs on DHBV. The 15 eZHBV sequences are provided in Dataset S1.

a GenBank accession number AY494851, isolated from a puna teal.

b Nonsense, stops/frameshifts.

c Distance to the nearest gene or exon.

**Figure 2:**
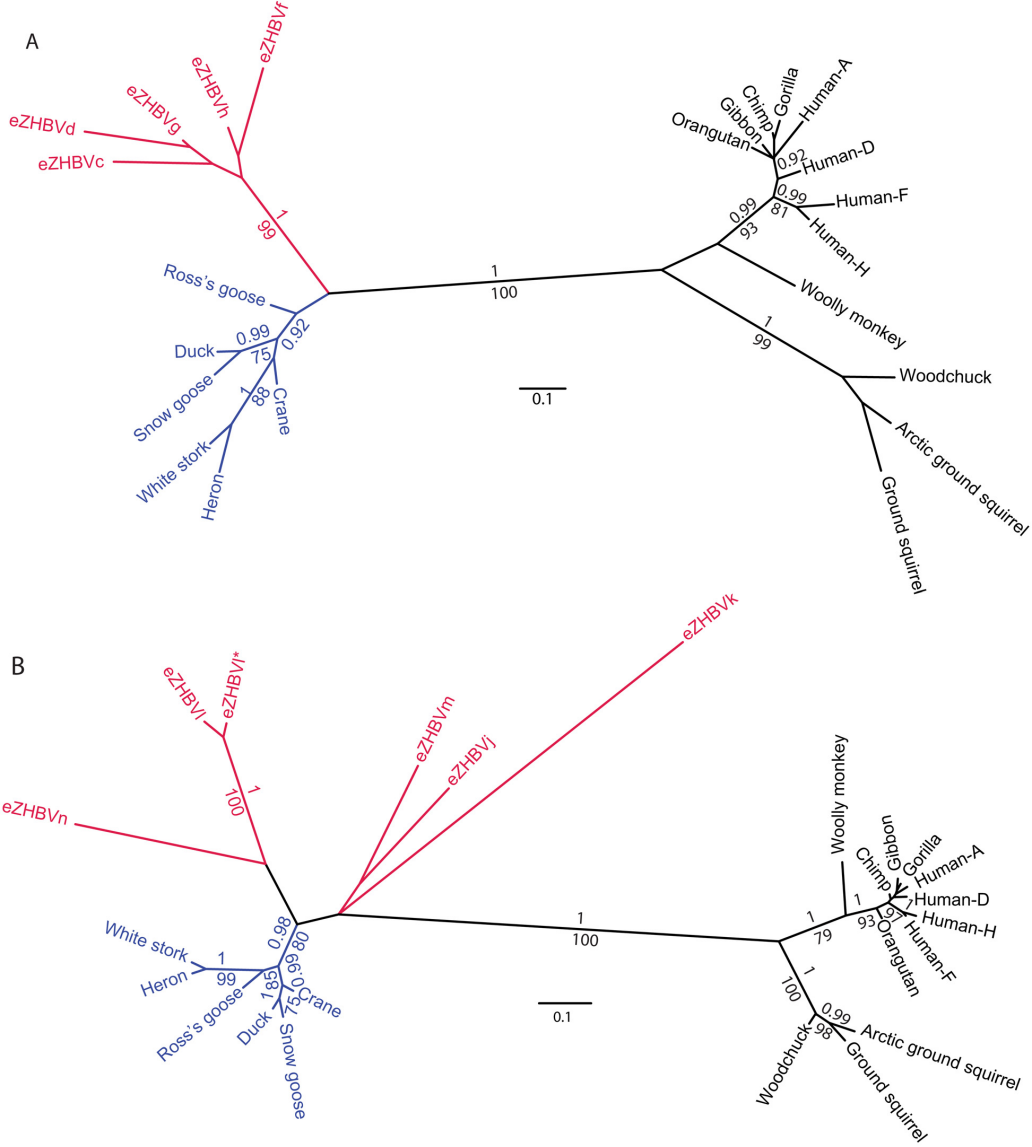
Unrooted trees of endogenous and extant hepadnaviruses. Avian hepadnaviruses are in red (endogenous) and blue (extant). The trees were obtained after maximum likelihood and Bayesian analyses of an amino acid alignment of two regions of the HBV genome: (A) from position 269 to 781 of DHBV, corresponding to group 1 sequences in Figure 1 (eZHBVe and eZHBVi were not included in the analyses because they overlap by only 46 bp) and (B) from position 1310 to 2521 of DHBV (GenBank accession number AY494851), corresponding to group 2 sequences in Figure 1. Numbers on the branches correspond to bootstrap values greater than 70 and posterior probabilities greater than 0.9. The alignments and accession numbers of the sequences are provided in Datasets S2 and S3.

In order to assess the phylogenetic relationship among eZHBVs and hepadnaviruses, we conducted phylogenetic analyses of amino acid alignments including extant hepadnaviruses and group 1 (106 amino acids) and group 2 (293 amino acids) eZHBVs. The results show that in both phylogenies (Figure 2A and 2B) hepadnaviruses can be divided into two clusters, one grouping eZHBVs and extant avian hepadnaviruses and the other including all mammalian hepadnaviruses. Within the former cluster, eZHBVs are consistently more distant from extant avian hepadnaviruses than these are from each other. While group 1 eZHBVs form a monophyletic group (Figure 2A), there is no statistically supported clustering of group 2 eZHBVs with each other (Figure 2B). The only exception is the close clustering of eZHBVl and eZHBVl*, which likely reflects their relatively recent origin by duplication rather than as independent insertions (see above).

### How Old Are eZHBVs?

A first minimal estimate of the age of eZHBVs can be derived indirectly from the time at which the duplication yielding eZHBVl and eZHBVl* occurred, which must postdate the chromosomal integration of the ancestral eZHBVl element. The distance between these duplicates is 0.03 (Table 2). To our knowledge, the most comprehensive estimate of neutral nuclear substitution rates available for birds, calculated based on a comparison of multiple intron sequences between chicken and turkey, was found to range between 2×10^−9^ and 3.9×10^−9^ substitutions per site per year (subs/site/year) [21], values similar to the range of those estimated for mammals (2.2×10^−9^ to 4.5×10^−9^ subs/site/year; [22],[23]). The avian rates are based on a fossil calibration of the split between Anatidae and Anhimidae at 55 My [21],[24],[25]. Dividing half of the distance between eZHBVl duplicates (0.015) by the bird neutral substitution rates yields a duplication time ranging between 3.8 My (with 3.9×10^−9^ subs/site/year) and 7.5 My (with 2×10^−9^ subs/site/year). The timing of this duplication provides a minimal estimate for the integration of the ancestral eZHBV fragment.

**Table 2:** Corrected distances between avian hepadnaviruses calculated on the region corresponding to group 2 eZHBVs (see Figure 1).

	eZHBVl	eZHBVl*	eZHBVj	eZHBVk	eZHBVn	eZHBVm	Crane	Ross's Goose	Heron	Snow Goose	White Stork
eZHBVl*	0.03										
**eZHBVj**	**0.89**	**0.87**									
**eZHBVk**	**1.99**	**1.77**	**1.51**								
**eZHBVn**	**0.55**	**0.58**	**0.81**	**2.43**							
**eZHBVm**	0.48	0.49	0.35	**1.43**	**0.84**						
**Crane**	0.49	0.46	0.81	1.53	0.49	0.42					
**Ross's goose**	0.44	0.42	0.80	1.51	0.48	0.41	0.17				
**Heron**	0.53	0.51	0.84	1.60	0.52	0.43	0.23	0.23			
**Snow goose**	0.47	0.45	0.76	1.46	0.49	0.44	0.14	0.17	0.22		
**White stork**	0.53	0.50	0.83	1.59	0.51	0.45	0.23	0.23	0.11	0.22	
**Duck**	0.51	0.49	0.77	1.43	0.48	0.43	0.14	0.18	0.23	0.09	0.26

Distances were calculated using the TVM+G model under maximum likelihood settings (see Materials and Methods) in PAUP [65]. Values in bold correspond to distances between eZHBVs that are larger than 0.54. This distance threshold corresponds to twice the average distance between extant avian hepadnaviruses (2×0.19 = 0.38) plus 0.16, which corresponds to a conservatively high estimate of the distance accumulated at the bird genome rate after integration (see Materials and Methods). eZHBVl and eZHBVl* derive from post-insertional duplication (see Results for details).

**Figure 3:**
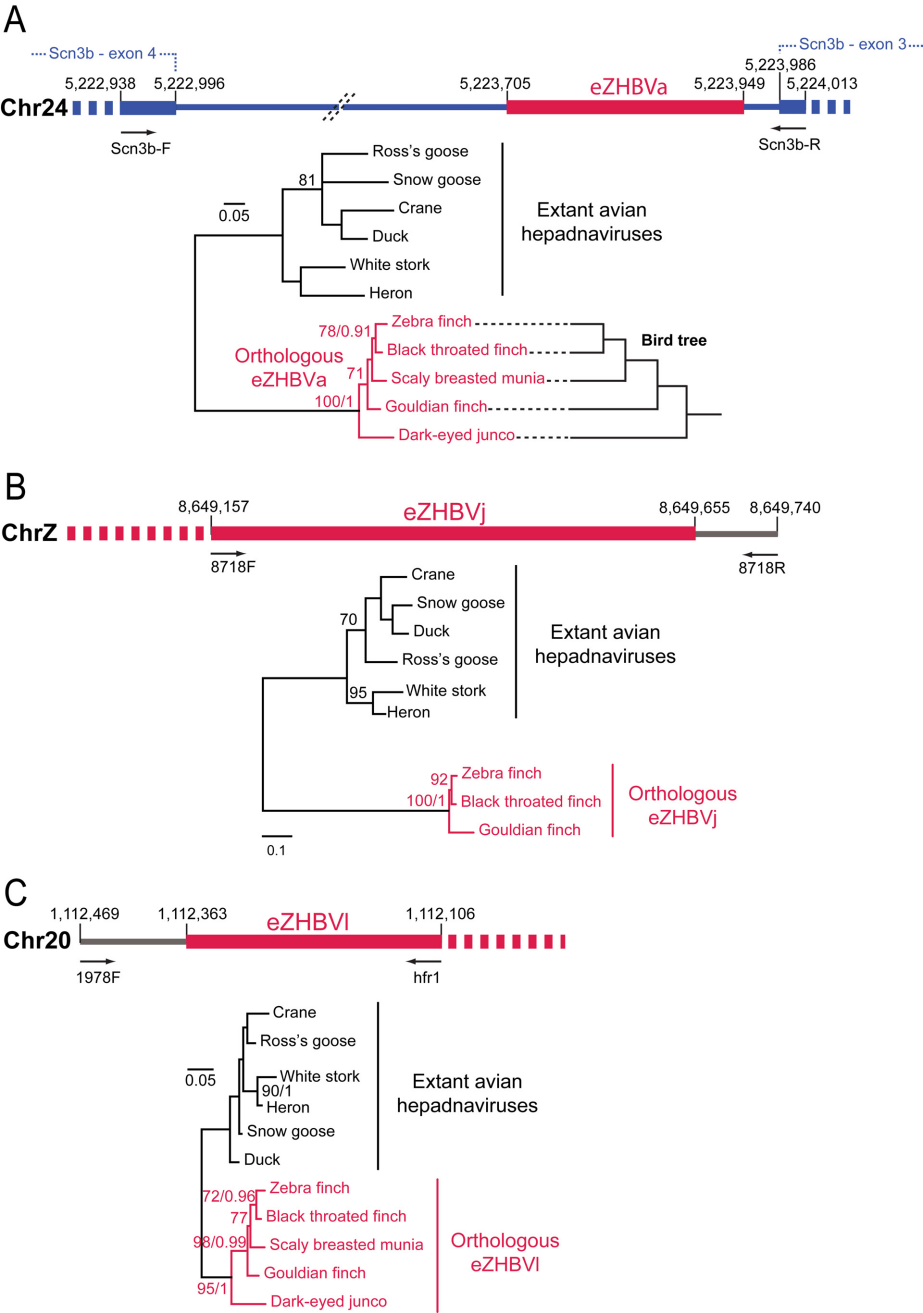
Illustration and phylogenetic trees of orthologous eZHBVa, eZHBVl, and eZHBVj. The primers (Scn3b-F/R) used to amplify eZHBVa (A) are anchored in exons 3 and 4 of a predicted gene homologous to the human *SCN3B* gene (blue), on zebra finch Chromosome 24. One of the primers (8718R) used to amplify eZHBVj (B) is located in the region flanking the insertion in 3′ on zebra finch Chromosome Z, while the other (8718F) is anchored in eZHBVj. One of the primers (1978F) used to amplify eZHBVl (C) is located in the region flanking the insertion in 5′ on zebra finch Chromosome 20, while the other (hfr1) is anchored in eZHBVl. Each orthologous eZHBV tree reflects the bird tree, derived from [28],[30] and illustrated in (A). The congruence between orthologous eZHBV trees and the bird tree is in each case consistent with one event of eZHBV integration in a common ancestor of the different birds where the insertion was found. The eZHBV trees are rooted using circulating avian hepadnaviruses as an outgroup. Numbers on branches correspond to bootstrap values greater than 70 and posterior probabilities greater than 0.9. The precise position and sequence of the PCR primers for each locus is given in Datasets S4, S5, S6. The chromosomal coordinates are derived from the July 2008 assembly of the zebra finch genome (WUGSC 3.2.4/taeGut1).

**Figure 4:**
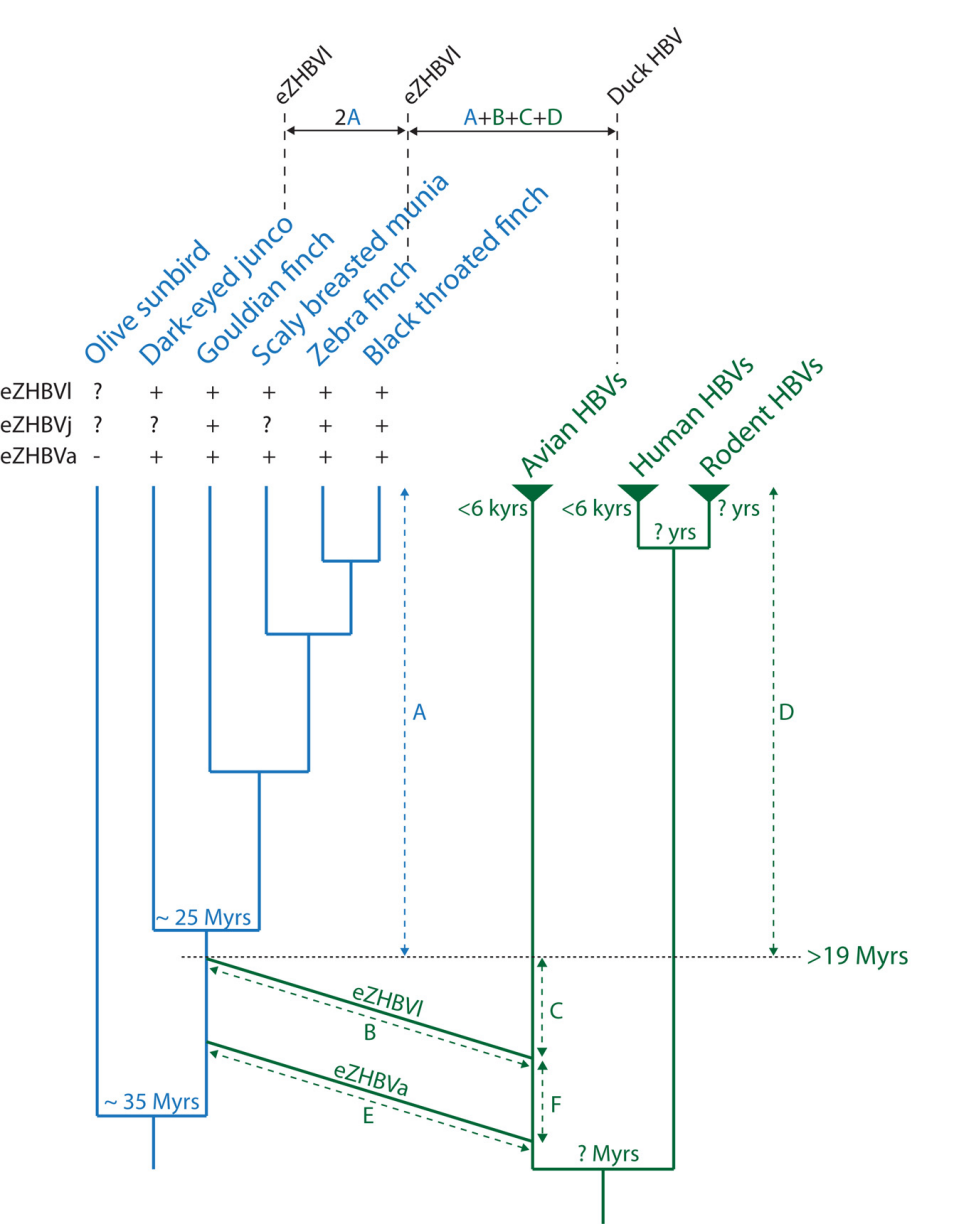
Summary of the evolutionary scenario inferred in this study. The bird tree (in blue, left) includes representatives of three families of passerine birds: Nectariniidae (olive sunbird, *C. olivaceus*), Emberizidae (dark-eyed junco, *J. hyemalis*), and Estrildidae (scaly breasted munia, *L. punctulata*; gouldian finch, *C. gouldiae*; zebra finch, *T. guttata*; and Black throated finch, *P. cincta*). Phylogenetic relationships between the different bird species are taken from [27],[28],[30]. Bird divergence times are taken from [29],[30]. The Hepadnaviridae tree (in green, right) is derived from Figure 2. The time for the most recent common ancestor of both human and avian extant hepadnaviruses has been estimated at less than 6,000 y [31]. The age of the ancestors of rodent and mammalian hepadnaviruses as well as that of the whole Hepadnaviridae family is unknown. The presence or absence of orthologous eZHBV insertions is denoted by “+” and “−”, respectively. A question mark indicates that it was not possible to determine whether the insertion was present or absent due to negative PCR. The germline infiltrations producing eZHBVl and eZHBVa are represented by the fusion of two branches of the hepadnavirus tree with the bird tree. Our conservative estimate of 19 My for the integration time of eZHBVa and eZHBVl is shown by a dash line (note that as eZHBVl could not be amplified in the olive sunbird, the time of integration of this fragment in the bird genome might predate the split between the olive sunbird and the Emberizidae + Estrildidae clade). The genetic distance between dark-eyed junco and zebra finch orthologous eZHBVl and that between the extant DHBV and eZHBVl are shown above the trees in order to illustrate the reasoning elaborated in the text. The former corresponds to the sum of the distance accumulated at the bird genome rate since integration (i) on the branch leading to the zebra finch and (ii) on the branch leading to the dark-eyed junco, i.e., 2×A. The latter corresponds to the sum of (i) the distance accumulated at the bird genome rate since integration (A), (ii) the distance accumulated at the viral rate since integration (D), and (iii) the distance accumulated at the viral rate between the time at which extant avian hepadnaviruses and eZHBVs diverged and the time of eZHBV endogenization (C + B for eZHBVl and F + E for eZHBVa). Note that C, F, E, and B are unknown and may be equal to zero.

A more direct way to estimate the age of eZHBVs is to use a phylogenetic approach, reasoning that if an insertion is shared by two species at the same (orthologous) locus, the integration event must be at least as old as the last common ancestor of the two species. It is important to note that the analysis of a large number of chromosomal integrants of HBV in somatic mammalian cells has revealed no preference for insertion in a specific sequence motif (e.g., [19],[26]). Thus, the possibility that two identical viral fragments would integrate at the exact same genomic position (i.e., between the same two nucleotides) independently in multiple species is extremely unlikely. Using PCR primers designed on the genomic regions flanking three eZHBVs, we were able to amplify two orthologous insertions (eZHBVa and eZHBVl) in three other species of estrildid finches (black throated finch [*Poephila cincta*], scaly breasted munia [*Lonchura punctulata*], and gouldian finch [*Chloebia gouldiae*]) and in the dark-eyed junco (*Junco hyemalis*), a non-estrildid passerine bird belonging to the Emberizidae family (Figure 3). We also obtained a positive PCR product for eZHBVj in the three estrildid finches, and were able to amplify the empty site orthologous to eZHBVa in the olive sunbird (*Cyanomitra olivaceus*, Nectariniidae) (Figures 3 and 4). The identity of all the eZHBV fragments amplified by PCR was confirmed by DNA sequencing (Datasets S4, S5, S6). This revealed that each orthologous eZHBV is present at the same chromosomal position in all species where it could be amplified. Furthermore, in all three cases, the phylogenetic relationships between orthologous eZHBVs reflect the phylogenetic relationships of the bird species (Figure 3). Together, these data strongly suggest that each of these three insertions descend from an ancestral integration event that occurred prior to the split of the different bird species.

The most recent molecular phylogenetic analyses divide finches and their allies into two major monophyletic clades, one consisting of African and Australasian estrildid finches and weavers, and the other grouping American emberizid sparrows (including the dark-eyed junco) together with fringillid finches and Old World sparrows [27]. Within Estrildidae, the gouldian finch is sister to a clade grouping the scaly breasted munia and finches of the genera *Poephila* (black throated finch) and *Taeniopygia* (zebra finch) (Figures 3A and 4; [28]). The congruence between these relationships and the phylogenies of orthologous eZHBVa and eZHBVl (Figure 3) indicates that the two eZHBVs result from two independent germline integration events of hepadnavirus-like sequences in a common ancestor of Estrildidae and Emberizidae, and that eZHBVa was inserted after the divergence of the Nectariniidae lineage. The divergence time between Estrildidae and Emberizidae has been estimated at 25 My based on relaxed molecular clock analyses of *rag1* and *rag2* nuclear genes using a paleobiogeographical calibration of 82 My for the split between Acanthisittidae and other passerine birds [29],[30]. The same analysis yielded an age of 35 My for the most recent common ancestor of Nectariniidae and Estrildidae. These dates would place the origin of eZHBVl prior to 25 My, and that of eZHBVa between 35 and 25 My ago.

Our last estimate of the age of eZHBVs relies on the level of sequence divergence between orthologous eZHBV sequences. The corrected distances inferred for orthologous eZHBVa (222 bp) and eZHBVl (238 bp) are 0.15 and 0.16 respectively between the zebra finch and the dark-eyed junco (see Materials and Methods). Selection analyses on these two fragments did not reveal any sign of positive or purifying selection (see Materials and Methods), suggesting that eZHBVa and eZHBVl have evolved under no functional constraint since their chromosomal integration in the common ancestor of these two birds, thereby accumulating substitutions at the neutral rate of these species. Applying the above-mentioned bird neutral substitution rates to half of the zebra finch/junco distances for eZHBVa and eZHBVl yielded integration times ranging between 40 My (with the eZHBVl distance of 0.08 and a rate of 2×10^−9^ subs/site/year) and 19.2 My (with the eZHBVa distance of 0.075 and a rate of 3.9×10^−9^ subs/site/year).

While our estimates of the age of eZHBVs are based on two different calibration points located at distant phylogenetic positions within the avian tree (55 My for the split between Anatidae and Anhimidae, or 82 My for the split between Acanthisittidae and other passerine birds), both approaches yield dates that largely overlap (40–19.2 My and 35–25 My). This suggests that eZHBVa and eZHBVl are at least 19 My old (and may be as much as 40 My old), which implies that the origin of avian hepadnaviruses as a whole (including extant and extinct viral lineages) is much deeper than the origin of currently circulating avian hepadnaviruses (time to most recent common ancestor <6,000 y; [31]).

### Long-Term Substitution Rates in Avian Hepadnavirus

Because eZHBVa and eZHBVl are at least 19 My old, the total genetic distance between these fragments and extant bird hepadnaviruses is expected to correspond to the sum of (i) the distance accumulated over the past 19 My at the bird neutral substitution rate (A in Figure 4), which can be approximated as half the distance between orthologous junco/zebra finch eZHBVa (0.075) or eZHBVl (0.08), (ii) the distance accumulated at the viral rate during the same period (D in Figure 4), and (iii) the distance accumulated at the viral rate between the time at which extant avian hepadnaviruses and eZHBVs diverged and the time of eZHBV endogenization (e.g., C+B for eZHBVl in Figure 4). The average corrected distance between eZHBVl and extant avian hepadnaviruses after subtracting the distance accumulated during 19 My at the bird rate (0.08) is 0.41 (range = 0.39–0.45). For eZHBVa, this distance is 1.3 (range = 1.15–1.5). Dividing these distances by 19 My yields average estimates of long-term substitution rates of 2.15×10^−8^ subs/site/year for eZHBVl and 6.8×10^−8^ subs/site/year for eZHBVa. Note that these values are likely to be overestimates as the distance between the time at which extant avian hepadnaviruses and eZHBVs diverged and the time of eZHBV endogenization is unknown (C+B for eZHBVl and F+E for eZHBVa, Figure 4), and therefore could not be subtracted from the total extant avian hepadnaviruses/eZHBV distance.

## Discussion

In this study we provide evidence that the germline genome of passerine birds has been infiltrated by several ancient and diverse hepadnaviruses that still show surprisingly high levels of similarity to extant avian hepadnaviruses. Although eZHBVs represent, to our knowledge, the first instance of endogenous DNA viruses reported in animals, several characteristics of hepadnaviruses suggest that endogenization of these viruses may be likely. Hepadnaviruses replicate in the nucleus of their host's cells via a reverse-transcribed RNA intermediate [32],[33]. Part of their life cycle is therefore spent in close proximity to the host DNA, which may facilitate chromosomal integration via various host- or transposable-element-mediated mechanisms that use either DNA or RNA templates (e.g., [15]). Indeed, although integration into the host genome is not required for the replication of the virus, integrated HBV genomic fragments are commonly observed in liver cells of individuals persistently infected, where they tend to be associated with hepatocarcinoma [19]. In addition, while hepadnavirus replication is thought to occur mainly in hepatocytes, its tropism may extend to other tissue and cell types, including germ cells. For example, avian hepadnavirus replication has been shown to occur in the yolk sac of developing duck embryos [34]. Typically, large quantities of viral particles circulate in the blood during HBV infection [35]. These particles have the capacity to tightly bind to many different cell types [35], and there is evidence supporting the presence of HBV DNA in spermatozoa and ovaries as well as the chromosomal integration of HBV in spermatozoa [36]–[38]. Based on these data, infiltration of the germline genome by hepadnaviruses followed by long-term vertical inheritance appears largely plausible. Thus, it is likely that other endogenous hepadnaviruses await discovery in other birds and perhaps also in mammalian genomes.

The precise mechanisms underlying the chromosomal integration of HBV remain unclear [19]. One model supported by experimental evidence posits that viral linear double-stranded DNA resulting from aberrant replication can be integrated during repair of double strand breaks via non-homologous end joining [39]. As the 3′ extremity of eZHBVj (position 2521) and eZHBVk (position 2512) map to a region of the DHBV genome that corresponds to the predicted end of a typical linear HBV precursor [40], the structure of these two fragments is potentially consistent with integration via non-homologous end joining. We also note that the extremities of several other fragments map to fairly narrow regions of the viral genome (e.g., same 5′ position for eZHBVd and eZHBVe; Figure 1), which may reflect the presence of breakpoint hotspots in the viral genomes that gave rise to eZHBVs. Finally, while the zebra finch genome contains several families of long terminal repeat (LTR) and non-LTR retrotransposons [41] whose enzymatic machinery could have potentially promoted the chromosomal integration of eZHBVs, none of the insertions examined were terminated by a poly-A tail or flanked by direct repeats, as would be expected if they had occurred through retrotransposition [15].

An intriguing question is whether the multiple eZHBVs result from endogenization events that took place during a short period of time or whether they were assimilated at widely different times over (at least) the past 19 My. Hepadnaviruses do not encode an integrase, and chromosomal integrants generally correspond to truncated genomes (as observed here). Thus, unlike retroviruses, integrated HBV fragments cannot in principle replicate further through intragenomic transposition or reinfection, and as such they can be considered essentially “dead on arrival.” With this in mind, we contend that eZHBVs are likely to result from multiple independent episodes of germline infiltrations that took place on a deep time scale, possibly spanning several millions of years, and involving distantly related hepadnaviruses. This inference is supported by the large distances observed between eZHBVs (Tables 2 and 3). Specifically, all pairwise distances involving eZHBVi and those between eZHBVl, eZHBVj, eZHBVk, and eZHBVn are more than 2-fold higher than the average distance separating extant avian HBVs, even when subtracting an approximate distance accumulated at the bird genome rate since integration (distances in bold in Tables 2 and 3). Together with the long branches leading to eZHBVs in the hepadnavirus tree (Figure 2), these data strongly suggest that diverse hepadnaviruses (at least five based on the distance threshold described above) have been circulating in birds for several million years. More specifically, we believe that the large inter-eZHBV distances likely reflect the fact that eZHBVs stem from viruses that were already deeply divergent at the time of integration, and/or that eZHBVs were integrated at time points separated by several million years over at least 19 My. A third, non-mutually exclusive explanation for these large distances is that the evolution of the hepadnavirus genome may be subject to strong mutational saturation (see also below). Considering that these viruses have crossed species boundaries repeatedly over the past 6,000 y [20],[31],[42], we speculate that a wide range of bird species may have been, and may still be, infected by hepadnaviruses. It would be interesting to explore whether hepadnaviruses are still circulating in extant estrildid finches such as the zebra finch. Such a discovery would provide a powerful system to study the virus and its potential association with hepatocarcinoma in a model bird species with a complete genome sequence [41].

**Table 3:** Corrected distances between avian hepadnaviruses calculated on the region corresponding to group 1 eZHBVs (see Figure 1).

	eZHBVc	eZHBVd	eZHBVe	eZHBVh	eZHBVi	eZHBVf	eZHBVg	Crane	Heron	Ross's Goose	Snow Goose	White Stork
**eZHBVd**	0.63											
**eZHBVe**	0.55	0.52										
**eZHBVh**	0.28	0.27	0.13									
**eZHBVi**	**2.45**	**2.05**	—	**1.07**								
**eZHBVf**	0.61	0.36	0.21	0.32	**2.07**							
**eZHBVg**	0.30	0.21	0.23	0.14	—	0.10						
**Crane**	1.04	1.13	1.46	0.57	1.13	0.95	0.62					
**Heron**	1.01	1.42	1.61	0.79	1.89	1.13	0.76	0.42				
**Ross's goose**	1.02	1.30	1.37	0.76	0.95	0.96	0.54	0.18	0.37			
**Snow goose**	1.03	1.16	1.55	0.70	1.00	1.10	0.71	0.17	0.39	0.20		
**White stork**	0.95	1.28	1.69	0.68	1.30	1.00	0.77	0.32	0.20	0.32	0.35	
**Duck**	1.09	1.34	1.11	0.66	1.20	1.07	0.61	0.18	0.40	0.16	0.09	0.32

Distances were calculated using the TPM2uf+G model under maximum likelihood settings (see Materials and Methods) in PAUP [65]. Values in bold correspond to distances between eZHBVs that are larger than 0.7. This distance threshold corresponds to twice the average distance between extant avian hepadnaviruses (2×0.27 = 0.54) plus 0.16, which corresponds to a conservatively high estimate of the distance accumulated at the bird genome rate after integration (see Materials and Methods).

Various calculations of HBV substitution rates based on comparison of extant viruses have produced broadly similar estimates, ranging from 7.72×10^−4^ to 7.9×10^−5^ subs/site/year [31],[43]–[47]. Surprisingly, we infer long-term substitution rates that are more than three orders of magnitude slower than these short-term rates. It is important to note that while eZHBVs evolved at the bird genome rate since their integration, this cannot explain the slowdown in long-term rates inferred in this study as the distance accumulated at the bird rate (A in Figure 4) was removed from our calculation of long-term hepadnavirus rates. Our estimates (2.15×10^−8^ to 6.8×10^−8^ subs/site/year) therefore represent a range of rates under which avian hepadnaviruses have evolved from the time just preceding the integration of eZHBVa and eZHBVl in the bird genome (∼19 My ago) to the time at which circulating avian hepadnavirus genomes were sequenced (the last two decades).

Gibbs et al. [48] recently suggested that viral evolutionary rates may vary dramatically depending on the time scale on which they are measured. The main line of evidence supporting this view was that rates inferred from serially or heterochronously sampled sequences are invariably more than two orders of magnitude higher than those calculated when assuming viruses have co-diverged with, and are therefore as old as, their hosts. In most cases, however, the hypothesis of host/virus co-divergence is only indirectly supported by the seemingly strong host specificity of the virus, and/or the apparent topological congruence (often not formally tested) between host and virus phylogenies. A major pitfall in this reasoning is that processes other than co-divergence may explain congruent phylogenies between hosts and viruses [49]–[51]. Given the potential caveats associated with the hypothesis of host/virus co-divergence, it is important to emphasize that our results do not rely on this assumption. Rather, they are based on a direct measure of the distance separating extant hepadnaviruses from extinct ones that are at least 19 My old.

How can we explain the apparent major disparity between short- and long-term substitution rates of hepadnaviruses? The rate of nucleotide substitution in any system depends on the background mutation rate, the rate of replication, and the rate of fixation. Hepadnaviruses replicate their genome via an RNA intermediate using a reverse transcriptase (RT). While to our knowledge there is no precise measure of the fidelity of the hepadnavirus RT, this enzyme lacks a proofreading activity and is known to be highly error prone in all retroviruses and other retroelements for which an error rate has been estimated [52],[53]. Up to 20-fold variations in RT error rates have been reported between different families of retroviruses [52]. It is therefore conceivable that variations in the fidelity of the enzyme (i.e., background mutation rate) over time might explain some of the difference between short- and long-term hepadnavirus substitution rates. However, slow long-term substitution rates similar to those reported here have been inferred for mammalian foamy viruses (1.7×10^−8^ subs/site/year) and human T cell lymphotropic virus type II (1.091×10^−7^ to 7.118×10^−7^ subs/site/year), two mammalian retroviruses that yet replicate via a highly error-prone RT [54],[55]. In those cases, it is thought that both viruses evolve slowly because they are non-pathogenic and replicate mainly as integrated proviruses, using the high-fidelity DNA polymerases of their hosts [56],[57]. These two examples therefore suggest that even in the presence of a high background mutation rate, viruses can evolve slowly if their replication rate is reduced. By analogy, it could be that hepadnaviruses have been characterized by low levels of pathogenicity and by low rates of replication for most of their evolutionary history. In this context, the high substitution rates and epidemiological dynamics currently associated with circulating hepadnaviruses might reflect recent drastic alterations in the biology of these viruses and of the selective pressures acting on them.

Another major process that may be responsible for the time dependency of substitution rates suggested by this study is purifying selection, as proposed for cellular organisms (e.g., [58]–[60]; see [61] for discussion). About 60% of the HBV genome codes for at least two overlapping open reading frames and therefore contains very few synonymous sites. Consistent with this, it was shown that nonoverlapping regions of the HBV genome evolve faster than overlapping regions [31],[62]. This tightly constrained genetic organization, combined with the intrinsically low fidelity of the RT, suggests that the effect of purifying selection on long-term rates may be more pronounced for hepadnaviruses than for other viruses and for cellular organisms. Lastly, the high background mutation rates of hepadnaviruses may also result in strong mutational saturation (homoplasy and back mutations), which could also explain part of the difference between short- and long-term hepadnavirus substitution rates (see also above). While it is possible that saturation may in part hinder our ability to accurately infer the long-term hepadnavirus substitution rates, we believe that this phenomenon alone cannot explain the 1,000-fold difference between short- and long-term substitution rates. Because our knowledge on the deep evolution of extant viruses remains fragmentary and because many factors may influence substitution rates and their variation over time [1],[63], it would be necessary to revisit these questions when more fossil and modern hepadnavirus sequences become available.

## Materials and Methods

### PCR and Sequencing

In order to screen for the presence or absence of orthologous eZHBVs in several species of passerine birds (Table S1), we designed PCR primers on the flanking regions of three insertions. The sequences produced using these primers were aligned and are provided, together with the sequence of the primers, in Datasets S5 (eZHBVl), S6 (eZHBVj), and S7 (eZHBVa). For eZHBVl, we used a forward primer (1978F) anchored in the 5′ flanking region (86 bp upstream of the insertion) in combination with a reverse primer (hfr1) anchored within eZHBVl, at position 239–257. For eZHBVj, we used a forward primer (8718F) anchored within eZHBVj at position 712–734 in combination with a reverse primer anchored in the 3′ flanking region (86 bp downstream of the insertion). For eZHBVa, we used a forward primer (Scn3b-F) anchored in the 5′ flanking region (768 bp upstream of the insertion in *T. guttata*) that corresponds to the fourth exon of a predicted gene homologous to human *SCN3B*. The reverse primer (Scn3b-R) was anchored in the 3′ flanking region (47 bp downstream of the insertion in *T. guttata*), corresponding to the third exon of the predicted *scn3b* gene.

The identity of the different bird species used in this study was verified by sequencing a 420-bp fragment of the mitochondrial NADH dehydrogenase subunit 2 (NADH2) gene (Figure S1) using the following primers: Fwd 5′–AGT CAT TTW GGS AGG AAT CCT G; Rev 5′–TTC CAY TTC TGA TTY CCA GAA G. Standard PCR conditions were as follows: 2 min at 94°C; 30 cycles of 1 min at 94°C, 30 s at 48–62°C, and 30 s to 2 min at 72°C. PCR mix was buffer (5×), 5 µl; MgCl2 (25 mM), 2 µl; dNTP (10 mM), 0.5 µl; primer 1 (10 µM), 1 µl; primer 2 (10 µM), 1 µl; Taq (GoTaq, Promega), 1.25 U; DNA, 30–100 ng; and H_2_O up to 25 µl. PCR products were directly sequenced on an ABI 3130XL sequencer (Applied Biosystems). All sequences produced in this study were submitted to GenBank (accession numbers HQ116564–HQ116583).

### Analyses of Selection

Analyses of selection were carried out on alignments of each set of orthologous insertions amplified in the various passerine birds (eZHBVl, eZHBVj, and eZHBVa; provided in Datasets S4, S5, and S6, respectively) using HyPhy [64]. We used the trees corresponding to each alignment as inferred in Figure 3. The nucleotide substitution model accomplishing the most accurate fit to the data was determined using the NucModelCompare.bf procedure: HKY85 for each of the three alignments. The MG94xHKY85_3x4 codon substitution model was then fitted to each alignment with global parameters and partition-based equilibrium frequencies. This yielded a global ω (non-synonymous substitutions/synonymous substitutions) ratio of 0.98 (confidence interval: 0.642323, 1.327), 0.66 (confidence interval: 0.44, 0.88), and 0.93 (confidence interval: 0.62, 1.24) for eZHBVl, eZHBVa, and eZHBVj respectively. Using a likelihood ratio test, the likelihood function states for each alignment were then compared to likelihood function states obtained using the same model/alignment/tree but enforcing ω = 1 (neutral evolution). This revealed no significant difference (*p* = 0.95 for eZHBVl, 0.16 for eZHBVa, and 0.81 for eZHBVj), suggesting that eZHBVl, eZHBVj, and eZHBVa are evolving neutrally. We further tested this by re-optimizing the likelihood function with local parameters (where each branch of the tree has its own parameters) and comparing the likelihood function state obtained when the non-synonymous substitution rate and the synonymous substitution rate can have their own value on each branch with the likelihood function state obtained when the non-synonymous substitution rate is forced to be equal to the synonymous substitution rate on each branch. Again, the likelihood ratio test revealed no significant difference (*p* = 0.61 for eZHBVl, 0.29 for eZHBVa, and 0.85 for eZHBVj), suggesting neutral evolution in all branches.

### Distances between Avian Hepadnaviruses

All distances were calculated under maximum likelihood settings in PAUP 4.0 [65], using models of nucleotide substitution chosen based on the Akaike Information Criterion in jModeltest [66]: TPM2uf+G for group 1 eZHBVs, TVM+G for group 2 eZHBVs and for the distance between eZHBVa and extant avian hepadnaviruses, TPM1 for the distances between passerine eZHBVa orthologs, and HKY for the distance between passerine eZHBVl orthologs.

In order to estimate whether eZHBVs result from multiple integrations of a few very similar viral strains during a narrow time frame or whether more divergent strains were endogenized at widely different times during the last 19 My, we compared inter-eZHBV distances to the average distances between extant avian hepadnaviruses. In this context, it is important to keep in mind that each pairwise inter-eZHBV distance as we observe them today results from (i) the distance accumulated at the viral rate during the time separating the endogenization of each two sequences being compared (corresponding to B+C+E+F if eZHBVl and eZHBVa are compared, for example; Figure 4) and (ii) the distance accumulated on each sequence at the bird neutral rate after endogenization (2×A in Figure 4). Several inter-eZHBV distances are more than 2-fold higher than the average distances between extant hepadnaviruses, i.e., more than 2×0.27 = 0.54 for the region corresponding to group 1 eZHBVs, and more than 2×0.19 = 0.38 for the region corresponding to group 2 eZHBVs (Tables 2 and 3). Notably, most of these high inter-eZHBV distances remain more than 2-fold higher than distances between extant hepadnaviruses even when subtracting a 0.16 distance, which corresponds to a conservatively high estimate of the distance accumulated at the bird genome rate assuming the two eZHBVs being compared were both integrated 19 My ago. The 0.16 estimate is based on the highest of the distances between dark-eyed junco and zebra finch orthologs (eZHBVl), i.e., 2×A in Figure 4.

### Phylogenetic Analyses

Sequences were aligned by hand using BioEdit 7.0.5.3 [67], and ambiguous regions were removed. Bayesian and maximum likelihood phylogenetic analyses were carried out using MrBayes 3.1.2 [68] and PHYML 3.0 [69], respectively. Nucleotide and amino acid substitution models were chosen based on the Akaike Information Criterion in jModelTest 0.1 [66], MrModeltest 2.3 [70], and ProtTest 2.4 [71]. eZHBVs were aligned at the amino acid level with representative members of extant avian and mammalian hepadnaviruses and analyzed using the rtREV (group 1 eZHBVs) and LG+G+F (group 2 eZHBVs) models in PHYML and with a prior setting allowing model jumping between fixed-rate amino acid models in MrBayes. eZHBVa, eZHBVj, and eZHBVl orthologs were analyzed with the TPM2uf+G, TPM2uf+G, and TIM3+G models of nucleotide substitution, respectively, in PHYML and with the GTR+G, HKY+G, and GTR+G models, respectively, in MrBayes. In order to verify the identity of the bird specimens included in this study, we also analyzed an alignment of a fragment of NADH2 nucleotide sequence produced in this study, as well as GenBank NADH2 sequences available for these species and for representatives of the families Paridae, Corvidae, Pycnonotidae, Turdidae, and Phasianidae (Figure S1). This alignment was analyzed with the TPM2uf+G model in PHYML and with the HKY+I+G model in MrBayes. For maximum likelihood analyses, the robustness of the branches was evaluated by non-parametric bootstrap analyses involving 1,000 pseudoreplicates of the original matrix. Bayesian analyses were run for at least one million generations, or until the standard deviation of split frequencies between the two parallel runs dropped below 0.01. Then, 25% of the sampled trees were discarded before summarizing the trees. The sequences used for the phylogenetic analyses are provided in Datasets S2, S3, S4, S5, S6, S7.

## Supporting Information

Dataset S1 **FASTA file containing the 15 eZHBVs found in the July 2008 assembly of the zebra finch genome (see also** **Table 1** **).**
(0.01 MB DOC)

Dataset S2 **Amino acid alignment (in FASTA format) of group 1 eZHBVs (see** **Figure 1** **) and representatives of known extant hepadnaviruses.** Ambiguous regions, stop codons, and frameshifts were removed. The names of the sequences include the GenBank accession numbers.
(0.00 MB DOC)

Dataset S3 **Amino acid alignment (in FASTA format) of group 2 eZHBVs (see** **Figure 1** **) and representatives of known extant hepadnaviruses.** Ambiguous regions, stop codons, and frameshifts were removed. The names of the sequences include the GenBank accession numbers.
(0.01 MB XLS)

Dataset S4 **Alignment of orthologous eZHBVl and 5′ flanking region (in FASTA format) sequenced in various passerine birds.** The 5′ end of eZHBVl corresponds to position 108 of the *T. guttata* sequence. The alignment includes the sequence of the primer 1978F, located in the 5′ flanking region of eZHBVl, and hfr1, located within eZHBVl.
(0.00 MB XLS)

Dataset S5 **Alignment of orthologous eZHBVa and 5′ and 3′ flanking regions (in FASTA format) sequenced in various passerine birds.** The 5′ and 3′ ends of eZHBVa correspond to positions 769 and 1012, respectively, of the *T. guttata* sequence. Positions 504–805 of the *J. hyemalis* sequence correspond to an endogenous retrovirus solo LTR (closely related to the zebra finch TguERVK9_LTR2g element; [41]) inserted within the region orthologous to eZHBVa. The solo LTR is flanked by a 6-bp target site duplication (GACCTT). The alignment includes the sequence of the primer Scn3b-F, located in the 5′ flanking region of eZHBVa, which corresponds to the fourth exon of a predicted gene homologous to human *SCN3B*, and that of the primer Scn3b-R, located in the 3′ flanking region of eZHBVa, which corresponds to the third exon of the predicted *scn3b* gene. Positions 1–59 and 1013–1076 of the *T. guttata* sequence correspond respectively to the partial sequence of the fourth and third exon of the predicted *scn3b* gene.
(0.01 MB XLS)

Dataset S6 **Alignment of orthologous eZHBVj and 3′ flanking region (in FASTA format) sequenced in various passerine birds.** The 3′ end of eZHBVj corresponds to position 1209 of the *T. guttata* sequence. The alignment includes the sequence of the primer 8718F, located within eZHBVj, and 8718R, located in the 3′ flanking region of eZHBVj.
(0.01 MB XLS)

Dataset S7 **Alignment (in FASTA format) of the NADH2 partial sequences used to construct the tree in Figure S1.**
(0.01 MB XLS)

Figure S1 **Phylogenetic tree of NADH2 sequences.** Numbers on branches correspond to bootstrap values and posterior probabilities. For most species, there is strong support grouping the sequence produced in this study and a NADH2 sequence of the same species available in GenBank, confirming the identification of the specimens from which the tissues used in this study come. The absence of support for the grouping of our *P. cincta* and that found in GenBank is due to the fact that the GenBank sequence is partial (Dataset S7). Phylogenetic analysis of a reduced alignment including only the NADH2 portion corresponding to the GenBank *P. cincta* sequence yields strong support for the grouping of the sequence obtained in this study with that in GenBank (bootstrap = 99, posterior probability = 1; data not shown). There is no NADH2 sequence available for *L. punctulata* in GenBank. While there is no support for the precise position of our *L. punctulata* sequence, we note that it tends to group with that of a congeneric species (*L. cucullata*).
(0.07 MB DOC)

Table S1 **Tissue samples used in this study.** All Estrildidae species were provided by the University of Washington Burke Museum.The *C. olivaceus* DNA was provided by Drs. Claire Loiseau and Ravinder Sehgal (San Francisco State University). The *J. hyemalis* tissue was sampled from a dead specimen found in CG's backyard in Arlington, Texas.
(0.03 MB DOC)

## Abbreviations

DHBV: duck hepatitis B virus
eZHBV: endogenous zebra finch hepatitis B virus
HBV: hepatitis B virus
LTR: long terminal repeat
NADH2: NADH dehydrogenase subunit 2
RT: reverse transcriptase
subs/site/year: substitutions per site per year
