# Supplementary figures and images for: Genomic Fossils Calibrate the Long-Term Evolution of Hepadnaviruses
Source: PLoS Biol. 2010 Sep 28;8(9):e1000495. doi: 10.1371/journal.pbio.1000495 (PMC2946954; doi:10.1371/journal.pbio.1000495)

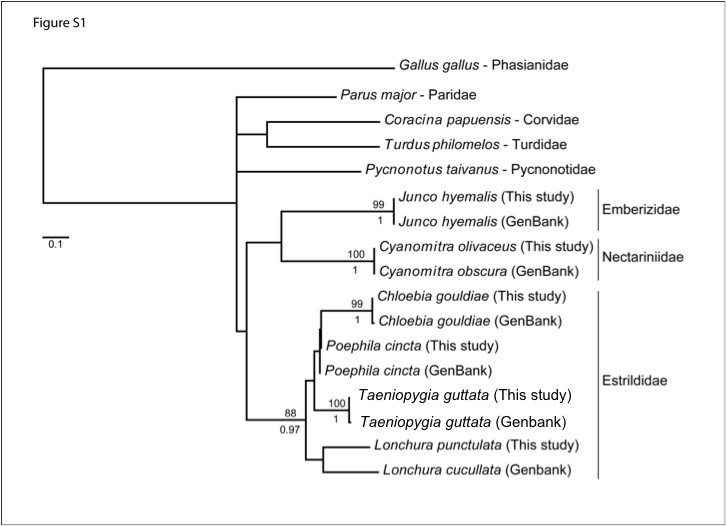

Supplement: Figure S1 — Phylogenetic tree of NADH2 sequences. Numbers on branches correspond to bootstrap values and posterior probabilities. For most species, there is strong support grouping the sequence produced in this study and a NADH2 sequence of the same species available in GenBank, confirming the identification of the specimens from which the tissues used in this study come. The absence of support for the grouping of our P. cincta and that found in GenBank is due to the fact that the GenBank sequence is partial (Dataset S7). Phylogenetic analysis of a reduced alignment including only the NADH2 portion corresponding to the GenBank P. cincta sequence yields strong support for the grouping of the sequence obtained in this study with that in GenBank (bootstrap = 99, posterior probability = 1; data not shown). There is no NADH2 sequence available for L. punctulata in GenBank. While there is no support for the precise position of our L. punctulata sequence, we note that it tends to group with that of a congeneric species (L. cucullata). (0.07 MB DOC) [file pbio.1000495.s008.jpg]
