# Supplementary material for: Genomic Fossils Calibrate the Long-Term Evolution of Hepadnaviruses
Source: PLoS Biol. 2010 Sep 28;8(9):e1000495. doi: 10.1371/journal.pbio.1000495 (PMC2946954; doi:10.1371/journal.pbio.1000495)
Supplement: Table S1 — Tissue samples used in this study. All Estrildidae species were provided by the University of Washington Burke Museum.The C. olivaceus DNA was provided by Drs. Claire Loiseau and Ravinder Sehgal (San Francisco State University). The J. hyemalis tissue was sampled from a dead specimen found in CG's backyard in Arlington, Texas. (0.03 MB DOC) [file pbio.1000495.s009.doc]

| **Family** | **Genus** | **Species** | **Voucher number** | **Accession** | **Location** | **Year of sampling** |
| --- | --- | --- | --- | --- | --- | --- |
| Estrildidae | *Poephila* | *guttata* | UWBM 60818 | 1998-068 | Australia, Western Australia, Fitzroy Crossing | 1997 |
| Estrildidae | *Poephila* | *cincta* | UWBM 57528 | 1996-109 | Australia, Queensland, Chillagoe | 1996 |
| Estrildidae | *Chloebia* | *gouldiae* | UWBM 84155 | 2006-001 | Captive, Seattle, Washington | 2005 |
| Estrildidae | *Lonchura* | *punctulata* | UWBM 83618 | 2006-117 | Singapore, Pasir Ris | 2006 |
| Emberizidae | *Junco* | *hyemalis* | - | - | USA, Texas, Arlington | 2009 |
| Nectariniidae | *Cyanomitra* | *olivaceus* | 271034 | - | Ghana | 2007 |
